# The Public Stigma of Problem Gambling: Its Nature and Relative Intensity Compared to Other Health Conditions

**DOI:** 10.1007/s10899-015-9580-8

**Published:** 2015-10-20

**Authors:** Nerilee Hing, Alex M. T. Russell, Sally M. Gainsbury, Elaine Nuske

**Affiliations:** Centre for Gambling Education and Research, Southern Cross University, PO Box 157, Lismore, NSW 2480 Australia

**Keywords:** Public stigma, Problem gambling, Gambling disorder, Societal stigma, Mental health, Treatment-seeking, Australia

## Abstract

Problem gambling attracts considerable public stigma, with deleterious effects on mental health and use of healthcare services amongst those affected. However, no research has examined the extent of stigma towards problem gambling within the general population. This study aimed to examine the stigma-related dimensions of problem gambling as perceived by the general public compared to other health conditions, and determine whether the publicly perceived dimensions of problem gambling predict its stigmatisation. A sample of 2000 Australian adults was surveyed, weighted to be representative of the state population by gender, age and location. Based on vignettes, the online survey measured perceived origin, peril, concealability, course and disruptiveness of problem gambling and four other health conditions, and desired social distance from each. Problem gambling was perceived as caused mainly by stressful life circumstances, and highly disruptive, recoverable and noticeable, but not particularly perilous. Respondents stigmatised problem gambling more than sub-clinical distress and recreational gambling, but less than alcohol use disorder and schizophrenia. Predictors of stronger stigma towards problem gambling were perceptions it is caused by bad character, is perilous, non-recoverable, disruptive and noticeable, but *not* due to stressful life circumstances, genetic/inherited problem, or chemical imbalance in the brain. This new foundational knowledge can advance understanding and reduction of problem gambling stigma through countering inaccurate perceptions that problem gambling is caused by bad character, that people with gambling problems are likely to be violent to other people, and that people cannot recover from problem gambling.

## Introduction

Recreational gambling is a popular and normalised activity in many societies. In contrast, problem gambling appears to attract considerable public stigma, resulting in deleterious effects on the health and use of treatment services amongst those affected. Public stigma is the reaction of society to people with a stigmatising condition and the formation of negative attitudes towards the stigmatised population (Corrigan 2004). It occurs when a negative attribute is publicly perceived, with those affected then judged, labelled and devalued, and either discredited if their stigmatising condition is known, or discreditable if hidden (Fernandez Y-Garcia et al. 2012; Goffman 1963). Public stigma therefore strengthens the division between those perceived as ‘normal’ and ‘others’ who are not (Rusch et al. 2005). Public stigma is thought to be particularly damaging for the health and wellbeing of stigmatised individuals. As well as facing stereotyping, prejudice and discrimination, they may experience the mental health effects of diminished self-worth and self-efficacy, withdraw from social support, and reject treatment and other interventions if they internalise publicly stigmatising beliefs as self-stigma (Corrigan and Watson 2002a, b). Stigma can also impact negatively on adjustment and growth, compromising mental wellbeing (Mak et al. 2007).

Problem gambling appears to be publicly stigmatised, although research has been largely confined to university student samples, which are not representative of larger populations (Gainsbury et al. 2014). Horch and Hodgins (2008) surveyed 249 undergraduate students to ascertain their desired social distance from a protagonist described in vignettes for five health conditions. ‘Disordered gambling’ was more stigmatised than normal sub-clinical worries and cancer, but similarly stigmatised as alcohol disorder and schizophrenia. A study with 281 university students found that ‘pathological gambling’ was the 13th most stigmatised amongst 40 mental illnesses, slightly less than alcohol dependence (rated 10th), more than paranoid schizophrenia (20th), and similar to substance-induced dementia (12th; Feldman and Crandall 2007). ‘Problem gamblers’ have been stereotyped as compulsive, impulsive, desperate, irresponsible, risk-taking, depressed, greedy, irrational, antisocial, and aggressive (Horch and Hodgins 2013). Even other frequent gamblers stigmatise people with gambling problems (Carroll et al. 2013). However, the public stigma associated with problem gambling has not been measured in general population samples (Hing et al. 2014).

Public stigma is apparent through its effects, especially on use of healthcare services. Stigma commonly deters individuals from acknowledging problems for fear of self-identifying as ‘a problem gambler’ (Hing et al. 2012; Suurvali et al. 2009). Many people keep gambling problems hidden to avoid social rejection through disclosing their ‘spoiled identity’ (Goffman 1963; Hing et al. 2014). The shame associated with having a gambling problem, the self-stigma of admitting it, fear of public stigma once disclosed, and stigma of attending treatment can all delay and deter treatment-seeking (Hing et al. 2014). Stigma is the most cited reason for avoiding professional treatment for mental health problems, including problem gambling (Corrigan 2004; Gainsbury et al. 2014; Rockloff and Schofield 2004; Tavares et al. 2002). Stigma reduction measures are needed to reduce negative health expectancies for stigmatised individuals and to improve treatment-seeking and recovery from problem gambling.

Developing appropriate stigma reduction initiatives requires understanding why a condition attracts societal stigma. Major theories of mental illness stigma suggest a condition’s perceived dimensions determine whether and how much it is publicly stigmatised (Corrigan et al. 2003; Jones et al. 1984; Weiner 1986). Thus, understanding how certain dimensions of problem gambling are perceived, and their relative contribution to its public stigmatisation, can inform strategies to counter misperceptions, inaccurate stereotypes, prejudice and discrimination, and encourage uptake of interventions and treatment. Therefore, this study aimed to (1) examine the stigma-related dimensions of problem gambling as perceived by the general public, (2) compare the stigma-related dimensions of problem gambling to those for other health conditions, and (3) determine whether the publicly perceived dimensions of problem gambling predict its public stigmatisation. Addressing these aims should advance knowledge of how problem gambling is viewed by society in terms of its dimensions and relative to other health conditions, and of the causes of its stigmatisation, to inform stigma reduction strategies that reduce related health impacts.

## Dimensions That Can Influence Public Stigma

There is no relevant theory as to why problem gambling attracts public stigma. However, two main theories explain why mental illness attracts public stigma. *Attribution theory* premises that the perceived *origin* of a stigmatising condition determines affective and behavioural responses towards stigmatised individuals and expectations about their future recovery (Weiner 1986; Weiner et al. 1988). External attributions (e.g., accident, genetic cause) should prompt sympathy, pity and helping behaviours, while internal attributions (e.g., lack of self-control, poor decision-making) usually elicit anger, annoyance and punishing behaviours. Greater stigma is expected when a condition’s origin is attributed to an individual’s personal actions rather than uncontrollable causes (Weiner 1986). Thus, individuals with mental illness are judged more harshly than those with physical disability, being perceived as having more personal responsibility for their condition (Corrigan et al. 2003; Socall and Holtgraves 1992; Weiner et al. 1988). Addictions are more negatively judged than other mental illnesses because those affected are considered more blameworthy for their disorder, and more dangerous (Angermeyer and Dietrich 2006).

Problem gambling appears to be attributed mainly to personal shortcomings. Carroll et al.’s (2013) interviewees viewed problem gambling as due to lack of self-control, absence of guilt, risk-taking propensity, ignorance of gambling odds, and unrealistic beliefs about winning. ‘Personal responsibility’ was one of three dimensions predicting stigmatisation of mental disorders, including pathological gambling (Feldman and Crandall 2007). University students considered stressful life circumstances and ‘bad character’ as the main causes of problem gambling (Dhillon et al. 2011; Horch and Hodgins 2008). While these studies provide important insights, their small student samples limit generalisability.

A second explanation for mental illness stigma, the *danger appraisal hypothesis* (Corrigan et al. 2003), accounts for a fear response to stigmatising attributes. Perceived *peril* elicits fear and desire for social distance, regardless of perceived origin (Corrigan et al. 2003). However, people experiencing problem gambling are not considered particularly dangerous, although desired social distance increased with higher perceived likelihood of violence (Dhillon et al. 2011; Horch and Hodgins 2008). Perceived dangerousness was one of three dimensions predicting stigmatisation of mental illnesses, including pathological gambling (Feldman and Crandall 2007).

Other perceived attributes have been proposed as contributing to societal stigmatisation of mental illness. One is *course*, with non-recoverable conditions tending to attract greater stigma than recoverable conditions (Jones et al. 1984). *Concealability* can also influence public stigma (Jones et al. 1984). Keeping a gambling problem hidden is common due to shame, embarrassment and fear of stigma, although this also hinders access to treatment, interventions and other support (Hing et al. 2012; Hodgins and el-Guebaly 2000). *Aesthetics* (Jones et al. 1984) may not be a stigmatising dimension for problem gambling given that it is not accompanied by any physical mark. However, the *disruption* caused by problem gambling to the lives of gamblers and significant others (Holdsworth et al. 2013) appears likely to contribute to its public stigmatisation.

Overall, these theories identify several dimensions that can contribute to the public stigmatisation of a condition. This study clarifies their role in the public stigmatisation of problem gambling.

## Methods

### Participants

A sample of 2000 adult residents of Victoria Australia was recruited through online panels from a market research company, based on quotas from the 2011 Australian Census (ABS 2011) for age (in brackets), sex and location of residence (Greater Melbourne and rest of Victoria). Younger male respondents were slightly difficult to recruit so quotas were relaxed towards the end of the survey period. After weighting to correct for this, the sample was mostly female (51.5 %), with a mean age of 46.0 years (*SD* = 16.7) and 75.2 % resided in Greater Melbourne, as per the Census.

### Procedure

Ethics approval was gained through a university human research ethics committee. The survey was hosted online by Qualtrics in March 2014, with 3895 respondents starting the survey and 3539 completing it. Qualtrics discarded responses that were out of quota and, as a quality assurance and validation process, deleted surveys with evidence of “straight-lining” responses or which were completed very quickly. Median completion time for the final sample of 2000 respondents was 25.2 min.

### Vignettes

Five vignettes were modelled around those used previously (Horch and Hodgins 2008; Link et al. 1999), except for a recreational gambling vignette created to determine whether any observed stigma was related to gambling generally, rather than to problem gambling specifically. The other four vignettes were: problem gambling, alcohol use disorder, schizophrenia, and a sub-clinical distress control. Vignettes (Appendix) were modified slightly so that (a) time frames were standardised (the last year), (b) cues about other people judging the protagonist were removed, (c) they were more inclusive of DSM-5 criteria for each condition, and (d) ethnicity, education and gender were kept constant. Only a male protagonist was depicted because problem gambling more frequently occurs amongst men. While a limitation, restriction to one gender was necessary to maintain a manageable survey length and consistency amongst vignettes.

### Measures

Respondents rated the protagonists of each vignette on the following measures.

#### Origin

Based on the *Perceived Causes Scale* (Link et al. 1999), respondents were asked “How likely do you think it is that X’s situation is caused by …” in relation to six items (Table 1). Response options ranged from extremely unlikely (0) to extremely likely (4).

**Table 1:** Responses to the origin scale for problem gambling

Origin	Very unlikely (1)	Unlikely (2)	Neither likely nor unlikely (3)	Likely (4)	Very likely (5)	Mean (SD)
His bad character	22.5	29.2	31.7	14.4	2.3	1.45 (1.06)
A chemical imbalance in his brain	13.7	20.8	33.8	27.8	3.8	1.87 (1.08)
Stressful circumstances in his life	2.8	6.4	19.6	56.0	15.2	2.74 (0.89)
A genetic or inherited problem	19.9	24.9	30.6	22.5	2.0	1.62 (1.10)
God’s will	72.7	13.3	11.0	2.3	0.7	0.45 (0.83)
The way he was raised	12.2	20.7	34.0	29.9	3.2	1.91 (1.05)

Weighted percentage of respondents who replied with each response to ‘How likely do you think it is that Dan’s situation is caused by …’

#### Peril

Respondents were asked to rate “How likely is it that X would do something violent to other people?”, based on Horch and Hodgins’ (2008) *Perceived Dangerousness Item*. Response options were: extremely unlikely (0), unlikely, neither likely nor unlikely, likely, extremely likely (4). They were also asked how likely it was that X would do something violent to himself, with same response options.

#### Concealability/Noticeability

A single item asked: “How noticeable would X’s situation be to his family and friends if he hadn’t told them about it?”. Response options were: not at all noticeable (0), somewhat noticeable, moderately noticeable, very noticeable, extremely noticeable (5).

#### Course/Recoverability

This was measured using a single item: “How strongly do you agree or disagree that people can recover from X’s situation?” The response options were strongly disagree (0) to strongly agree (4).

#### Disruptiveness

Three items were selected from the *Key Informants Questionnaire*, a previously validated scale (e.g., Alem et al. 1999). Respondents were asked how much they thought the protagonist’s situation would affect his ability to live independently, be in a serious relationship, and work or study. Response options were not at all (0), small amount, moderate amount, large amount, extreme amount (4). Cronbach’s alpha for this scale was 0.77 for the problem gambling vignette and higher for all other vignettes, indicating acceptable reliability.

#### Separating

The 6-item *Social Distance Scale* (Martin et al. 2000) was used to measure stigma, with respondents rating their willingness to interact with the protagonist. Response options ranged from: definitely unwilling (0) to definitely willing (4). Cronbach’s alpha were between 0.85 and 0.90 for all vignettes.

### Design and Randomisation

All respondents were presented with the problem gambling and sub-clinical distress vignettes. The remaining vignettes were randomly allocated: alcohol use disorder (*n* = 672), schizophrenia (*n* = 633) and recreational gambling (*n* = 695). Thus, all participants saw just three vignettes to contain the overall length of the survey. The order of the vignettes was randomised for each respondent.

As randomisation does not ensure that each group is equal, responses to the measures on the common vignettes were compared between those allocated to each randomised vignette. The groups did not differ significantly on most measures. Where differences were found, effect sizes were very small and most likely only significant due to the large sample size. We therefore reported pooled statistics for the different groups for the common vignettes.

### Data Weighting

Weighting corrected for differences between the sample and the 2011 Australian Census, and were calculated based on a cross-tabulation of gender, age (18–29, 30–39, 40–49, 50–59, 60–64, 65+) and location of residence, using an iterative procedure. The final weights ranged between 0.62 and 2.20, indicating no extreme weights and mild effects on the final results. These weights were applied for all analyses.

### Data Analysis

Repeated measures analyses compared responses to the problem gambling vignette to responses to the other vignettes. The randomised vignettes had different sample sizes and the associated analyses therefore have different power. The reported effect sizes should be considered when interpreting the results. A multiple linear regression was conducted to examine Aim 3. As response scales were Likert scales, we treated the data as continuous and used parametric statistics. Finally, an estimation of the proportion of respondents who stigmatised each condition was calculated using the Separating scale, as described in the relevant section of the results. McNemar tests were used to compare these proportions.

Respondents were also asked about their exposure to problem gambling, using a modified version of the Level of Contact report (Holmes et al. 1999). A weak correlation was observed between level of exposure and the Separating scale (*r* = 0.11), such that those who had more exposure to problem gambling were more likely to socialise with the person in the problem gambling vignette. Controlling for this exposure effect made no difference to the present results. As such, the relationship between exposure to problem gambling and stigma is not explored further in this paper.

## Stigma-Related Dimensions of Problem Gambling as Perceived by the General Public

Most respondents believed that the origin of the condition in the problem gambling vignette was likely or very likely due to stressful circumstances (71.2 %), but unlikely/very unlikely due to the person’s bad character (51.7 %) or God’s will (86.0 %). More respondents thought that problem gambling was unlikely/very unlikely to be due to a genetic or inherited problem (44.8 vs 24.5 % likely/very likely). Nearly equal proportions of respondents thought that it was likely or unlikely that problem gambling was due to a chemical imbalance in the brain or the way the protagonist was raised. When mean scores were considered, respondents viewed problem gambling as most likely due to stressful life circumstances (smallest comparison vs other origins was *t*(1999) = 29.80, *p* < 0.001, *d* = 0.85), followed by the way the person was raised, chemical imbalance in the brain, genetic or inherited problem, bad character, and God’s will, respectively (Table 1).

In terms of peril to others, 22.9 % of respondents thought that it was likely (20.8 %) or very likely (2.1 %) that the protagonist would do something violent to other people, but 42.1 % thought this was unlikely (31.8 %) or very unlikely (10.3 %). However, 41.9 % indicating that it was likely (37.1 %) or very likely (4.8 %) that the person would harm himself, compared to 22.3 % indicating that this was unlikely (17.8 %) or very unlikely (4.5 %).

In relation to the course dimension, most respondents (81.6 %) agreed that people can recover from problem gambling (58.9 % agreeing; 22.7 % strongly agreeing). The vast majority considered that problem gambling was noticeable, with 95.2 % % stating it was a somewhat (23.3 %), moderately (30.2 %), very (32.4 %) or extremely (9.3 %) noticeable condition. When measured on the disruptiveness scale, most respondents indicated that problem gambling would have at least a large effect on ability to work or study (74.3 %), live independently (62.9 %), and be in a serious relationship (78.5 %).

## Stigma-Related Dimensions of Problem Gambling Compared to Those for Other Health Conditions

Table 2 presents the mean scores on each dimension for the problem gambling vignette, and the mean differences from and statistical comparisons to the problem gambling vignette for each of the other vignettes on each dimension.

**Table 2:** Statistical comparisons for each vignette compared to the problem gambling vignette for each scale

Vignette	Problem gambling (*n* = 2000)	Sub-clinical distress (*n* = 2000)			Alcohol use disorder (*n* = 680)			Schizophrenia (*n* = 630)			Recreational gambling (*n* = 690)		
	Actual	Difference			Difference			Difference			Difference		
Statistic	*M* (*SD*)	*M* (*SD*)	*t*	*d*	*M* (*SD*)	*t*	*d*	*M* (*SD*)	*t*	*d*	*M* (*SD*)	*t*	*d*
Origin—His bad character	1.45 (1.06)	−0.66 (1.09)	27.15*	1.21	−0.07 (1.00)	n.s.		−0.56 (1.04)	13.49*	1.08	−0.77 (1.05)	19.11*	1.52
Origin—A chemical imbalance in the brain	1.87 (1.08)	0.23 (1.33)	7.64*	0.34	0.35 (1.08)	8.33*	0.64	1.46 (1.22)	29.93*	2.39	−1.11 (1.21)	24.24*	1.93
Origin—Stressful circumstances in his life	2.74 (0.89)	0.33 (1.04)	14.07*	0.63	0.31 (0.90)	8.92*	0.69	0.07 (1.18)	n.s.		−1.29 (1.31)	25.87*	2.06
Origin—A genetic or inherited problem	1.62 (1.10)	0.23 (1.31)	7.81*	0.35	0.41 (1.05)	10.20*	0.78	1.02 (1.26)	20.30*	1.62	−0.78 (1.17)	17.56*	1.40
Origin—God’s will	0.45 (0.83)	0.08 (0.72)	5.24*	0.23	−0.01 (0.60)	n.s.		0.11 (0.69)	3.89*	0.31	−0.04 (0.71)	n.s.	
Origin—The way he was raised	1.91 (1.05)	−0.10 (1.22)	3.78*	0.17	0.24 (1.00)	6.27*	0.48	−0.43 (1.29)	8.38*	0.67	−0.37 (1.13)	8.54*	0.68
Peril to others	1.72 (0.97)	−0.67 (1.07)	27.90*	1.25	0.77 (0.98)	20.40*	1.57	0.83 (1.12)	18.58*	1.48	−1.32 (0.99)	35.02*	2.79
Peril to self	2.20 (0.94)	−0.65 (1.19)	24.39*	1.09	0.26 (1.02)	6.61*	0.51	0.67 (1.06)	15.88*	1.27	−1.70 (1.05)	42.58*	3.40
Course	0.97 (0.80)	0.14 (0.97)	6.41*	0.29	0.03 (0.69)	n.s.		−0.51 (0.97)	13.16*	1.05	0.35 (1.09)	8.42*	0.67
Concealability	2.18 (1.04)	−1.25 (1.21)	46.38*	2.08	0.51 (1.20)	10.99*	0.84	0.86 (1.31)	16.47*	1.31	−1.43 (1.29)	29.10*	2.32
Disruptiveness	2.85 (0.73)	−1.54 (0.96)	71.66*	3.21	−0.19 (0.73)	6.93*	0.53	0.43 (0.78)	13.75*	1.10	−2.36 (0.96)	64.81*	5.17

* indicates *p* < 0.001 (this critical *p* used to correct for multiple comparisons). n.s. indicates a difference that was not statistically significant. *d* indicates Cohen’s *d* and is only presented for statistically significant results. The means and SDs presented for the problem gambling vignette are for the whole sample. All vignettes were compared to this vignette on each scale (using related-sample *t* tests). The alcohol use disorder, schizophrenia and recreational gambling vignettes were not seen by all respondents and thus the mean for the problem gambling vignette for those comparisons is different to that shown here due to carryover effects. Therefore, the means and SDs for the vignettes that are not about problem gambling are *difference* scores

Compared to alcohol use disorder, problem gambling was perceived as significantly less likely to be due to a chemical imbalance in the brain, stressful life circumstances, a genetic or inherited problem, or the way the protagonist was raised. There was no significant difference between the two disorders for bad character or God’s will. Problem gambling was also perceived as significantly less perilous to self and others, less noticeable, but more disruptive than alcohol use disorder. No significant differences were observed between the two disorders for the course dimension.

Compared to schizophrenia, problem gambling was perceived as significantly less likely to be due to a chemical imbalance in the brain, a genetic or inherited problem, or God’s will, and significantly more likely to be due to bad character or the way the protagonist was raised. No significant differences were found between the two conditions for stressful life circumstances. Problem gambling was also perceived as significantly less perilous to self and others, less noticeable, less disruptive, but more recoverable, compared to schizophrenia.

Compared to sub-clinical distress, problem gambling was perceived as significantly less likely to be due to a chemical imbalance in the brain, stressful life circumstances, a genetic or inherited problem, or God’s will, but more likely to be due to bad character and upbringing. Problem gambling was also perceived as significantly more perilous to self and others, more disruptive and more noticeable, but less recoverable.

Compared to recreational gambling, problem gambling was perceived as significantly more likely to be due to all origins, except God’s will, where no significant difference was identified. Problem gambling was also perceived as significantly more perilous to self and others, more disruptive, more noticeable and less recoverable, compared to recreational gambling.

## Do the Publicly Perceived Dimensions of Problem Gambling Predict Its Public Stigmatisation?

Mean score on the social distance scale was 1.84 (SD = 0.74), reflecting a slight overall unwillingness to socialise with the problem gambling protagonist. Respondents showed a definite unwillingness to form a close, enduring relationship such as have the protagonist marry into the family (*M* = 0.99, *SD* = 0.87); some unwillingness to form a professional relationship such as working closely together (*M* = 1.80, *SD* = 1.00); but some willingness for more incidental social interaction such as moving next door (*M* = 1.94, *SD* = 1.02) or having a group household in their neighbourhood for people with problem gambling (*M* = 2.05, *SD* = 1.05), making friends with the protagonist (*M* = 2.07, *SD* = 0.97) or spending an evening socialising with him (*M* = 2.19, *SD* = 1.00).

A multiple linear regression determined which of the following factors were significant predictors of problem gambling stigma when controlling for each other: origin (six items), peril to others, concealability, course and disruptiveness. The dependent variable was the social distance scale, where higher scores indicate less desired social distance and, therefore, less stigma.

Initial analysis indicated no missing values. Independent variables were checked for high intercorrelations and correlations with the dependent variable. The highest intercorrelation amongst the independent variables was 0.50 (Table 3), between “a chemical imbalance in his brain” and “a genetic or inherited problem”. However, tolerance statistics indicated little problem with multicollinearity (lowest tolerance = 0.69) so all potential predictors were retained. All potential predictors were correlated with the DV, apart from some of the Origin items, notably “God’s will”. This particular item had little intra-item variability and was thus removed from the analysis. All other variables were retained, including the other Origin items, in order to gain a better understanding of all bivariate relationships. The assumptions of multiple linear regression were checked and all were satisfactory.

**Table 3:** Bivariate correlations between the dependent variable (social distance scale) and the independent variables for the multiple linear regression

Variable	Stigma (DV)	(1)	(2)	(3)	(4)	(5)	(6)	(7)	(8)
Origin—His bad character (1)	−0.305								
Origin—A chemical imbalance in his brain (2)	0.034^^^	0.107							
Origin—Stressful life circumstances (3)	0.066	0.076	0.200						
Origin—A genetic or inherited problem (4)	0.029^#^	0.154	0.499	0.123					
Origin—The way he was raised (5)	−0.089	0.337	0.202	0.186	0.307				
Peril to others (6)	−0.310	0.354	0.175	0.091	0.131	0.226			
Noticeability/concealability (7)	−0.158	0.153	0.077	0.044	0.041	0.070	0.214		
Course/recoverability (8)	0.237	−0.114	−0.080	0.073	−0.125	−0.071	−0.153	−0.094	
Disruptiveness (9)	−0.203	0.154	0.114	0.163	0.053	0.121	0.299	0.310	−0.039

The dependent variable is the social distance scale, where higher scores mean *less* stigma. Weights applied as per all other results. All correlations were statistically significant (<0.05) apart from ^^^ (*p* = 0.064) and ^#^ (*p* = 0.097)

Together, the model accounted for 20.9 % of variance in the dependent variable and this was significant, *F*(9, 1990) = 58.48, *p* < 0.001.

All predictors apart from the way he was raised were significant predictors of social distance/stigma. Those reporting stronger stigma were more likely to believe that the condition originated in his bad character, he would do something violent to other people, he cannot recover from problem gambling, and being a problem gambler is disruptive; *not* believe that problem gambling is due to stressful life circumstances, a genetic or inherited problem, or a chemical imbalance in his brain; and believe that the condition is more noticeable (Table 4).

**Table 4:** Coefficients from the multiple linear regression predicting stigma (social distance) for problem gambling, sorted by order of predictive strength

Predictor	Unstandardised coefficient (*SE*)	Standardised coefficient	*t*	*p*	95 % CI (LB: UB)
Origin—His bad character	−0.155 (0.016)	−0.220	−9.841	<0.001	(−0.185: −0.124)
Peril to others	−0.146 (0.017)	−0.190	−8.418	<0.001	(−0.180: −0.112)
Course/recoverability	0.170 (0.019)	0.183	8.926	<0.001	(0.133: 0.208)
Disruptiveness	−0.120 (0.022)	−0.118	−5.376	<0.001	(−0.164: −0.076)
Origin—Stressful life circumstances	0.070 (0.017)	0.084	4.008	<0.001	(0.036: 0.104)
Origin—A genetic or inherited problem	0.049 (0.016)	0.072	3.026	0.003	(0.017: 0.081)
Origin—A chemical imbalance in his brain	0.047 (0.016)	0.068	2.901	0.004	(0.015: 0.079)
Concealability	−0.030 (0.015)	−0.042	−1.976	0.048	(−0.060: 0.000)
Origin—The way he was raised	0.005 (0.016)	0.007	0.319	0.750	(−0.026: 0.036)

The dependent variable is the social distance scale, where higher scores mean *less* stigma. Weights applied as per all other results

## Estimated Proportion of the Public That Stigmatise Each Condition

It may be useful for policy makers and service providers to estimate the proportion of respondents who hold negative views of each of the conditions. In order to do so, the Separating measure for each of the vignettes was dichotomised, such that those who scored from 0 to 1.99 on a vignette were considered to be generally unwilling to socialise with the protagonist in that vignette, and those who scored 2 to 4 on the scale were considered neutral or willing to socialise with the protagonist. Using this measure, 51.7 % stigmatised problem gambling. A series of McNemar tests indicated that the proportion of respondents who stigmatised problem gambling was significantly lower than those who stigmatised alcoholism (59.1 %) and schizophrenia (52.6 %), but significantly higher than those who stigmatised recreational gambling (12.7 %) or sub-clinical distress (10.8 %; all *p* < 0.017).

## Discussion

This study has yielded new findings in two main areas. The first relates to how problem gambling is perceived by the general public in terms of the five dimensions examined (Corrigan et al. 2003; Jones et al. 1984; Weiner 1986). Stressful life circumstances was the only cause in the origin dimension endorsed by most respondents. This finding is consistent with previous studies examining the perceived origin of problem gambling, although unlike previous studies bad character was not commonly endorsed (Dhillon et al. 2011; Horch and Hodgins 2008). The use of student samples in previous studies, along with cultural differences, may explain this difference. In our survey, other commonly endorsed contributing factors to problem gambling were the way the person was raised, followed by a chemical imbalance in the brain. This finding aligns with the Pathways Model of Problem and Pathological Gambling (Blaszczynski and Nower 2002), specifically Pathway 2 gamblers, whose problematic gambling is motivated by a desire to modulate or escape negative emotional states such as stress, and whose emotional vulnerability has been exacerbated by negative childhood experiences.

Problem gambling was not perceived as particularly perilous to others. Fewer than one-quarter of respondents believed that the problem gambling protagonist was likely to be violent to others, in general alignment with previous studies (Dhillon et al. 2011; Horch and Hodgins 2008). Our survey found a stronger perception, endorsed by about two-fifths of respondents, that people with gambling problems are likely to do something violent to themselves, which accurately reflects this cohort’s heightened risk of suicide (Delfabbro 2012). Furthermore, a substantial majority recognised that problem gambling is highly disruptive, specifically endorsing large disruptions to ability to work or study, live independently, and be in a serious relationship. These findings suggest public recognition of the well-documented negative impacts that problem gambling typically has across personal, interpersonal, financial and vocational domains (Delfabbro 2012). This may be a result of media campaigns designed to encourage treatment-seeking that depict people with problem gambling as having severely disrupted lives. Over four-fifths of respondents considered problem gambling to be recoverable, reflecting a strong public perception that problem gambling can be resolved and is at least partially under personal control. These public perceptions appear to be accurate, given that recovery from problem gambling is common (Abbott et al. 2004; Slutske et al. 2009).

An unexpected finding was that the vast majority of respondents considered problem gambling to be at least a somewhat noticeable condition to family and friends, even if they had not been told about the person’s gambling problem, including over two-fifths considering it would be very or extremely noticeable. This finding contradicts research documenting the surprise and shock that most people report when informed about a significant other’s gambling problem, which has typically become severe before disclosure (Holdsworth et al. 2013; Patford 2008, 2009). This finding may reflect public underestimation of the secrecy typically accompanying problem gambling (Hing et al. 2012, Hodgins and el-Guebaly 2000). Raising public awareness of the signs of problem gambling may increase people’s capacity to recognise and respond to gambling problems amongst significant others, even if the latter have not yet disclosed the problem.

Further insights into the perceived nature of problem gambling can be gained from comparisons with other health conditions. Respondents perceived problem gambling as more debilitating than having normal sub-clinical worries and as distinct from recreational gambling. The effects of problem gambling were believed to be less severe than those of schizophrenia, with the former perceived as a developed condition in reaction to life circumstances rather than a predisposed condition beyond personal control. This contention was also supported by respondents’ overall views that problem gambling is just as likely to be caused by bad character as is alcohol use disorder, and is just as recoverable. However, the physical effects of heavy alcohol consumption and the resultant behavioural consequences likely explain why alcohol use disorder was perceived as more noticeable and perilous, compared to problem gambling.

Other similarities in how the five vignettes were perceived are informative, particularly in relation to perceived origin. Problem gambling, alcohol dependence and normal sub-clinical worries were perceived mainly as responses to life stressors, in contrast to a biological explanation for schizophrenia. Upbringing, along with stressful life circumstances, were believed to be the main contributors to recreational gambling. These two causes were also endorsed for problem gambling, but in reverse order, with stressful circumstances perceived as a much stronger contributor to problem gambling. These findings suggest that socialisation into gambling while growing up is viewed as largely shaping future gambling propensity, but that stressful circumstances are perceived to result in heavier gambling as a mechanism to cope with life’s pressures. Several studies have found that people exposed to gambling at an early age are more likely to gamble themselves and that people growing up with a problem gambling family member are more likely to develop problem gambling (Abbott and Volberg 1992; Dowling et al. 2010; Saugeres et al. 2012). Further, people brought up around gambling have been found to return to gambling, and to gamble problematically, when faced with stressful life events (Holdsworth et al. 2015). Thus, the perceived contributions of upbringing to recreational gambling and of stress to problem gambling appear to have some accuracy.

The second set of new findings is the contribution of the five dimensions to the public stigma of problem gambling. Support was found for attribution theory; that attributing a condition to a person’s own actions leads to greater stigma than when the cause is perceived as uncontrollable (Weiner 1986; Weiner et al. 1988). Problem gambling was more stigmatised when believed to be due to bad character, which may be considered a personal failing, rather than due to the external uncontrollable causes of stressful life circumstances, genetic or inherited problem, or chemical imbalance in the brain. Support was also found for the danger appraisal hypothesis (Corrigan et al. 2003), with greater stigma attached when believing that the problem gambling protagonist was likely do something violent to others. Support was also found for the other stigma-related dimensions (Jones et al. 1984), where desired social distance increased with the strength of belief that problem gambling was irrecoverable, disruptive and noticeable.

These findings can inform stigma reduction efforts to help improve health outcomes for people with gambling problems. The most stigmatising and inaccurate perceptions found were beliefs that problem gambling is caused by bad character, that affected people are likely to be violent to others, and that people cannot recover. While only minorities of respondents held these beliefs, countering these perceptions through community education and increasing community contact with people with gambling problems to challenge these assumptions should help reduce societal stigma of problem gambling. However, Corrigan and Fong (2014) caution that effective stigma-change interventions need to be distinguished from those which are less effective and from those which may have unintended consequences. They found that contact generally had superior effects to education in reducing public stigma, but that longer-term effects were unknown. Cook et al. (2014) also emphasise the synergistic value of multi-level initiatives to reduce stigma and its health consequences, that target the stigmatised group, the non-stigmatised group and the socio-political environment.

The dimensions examined explained only one-fifth of the variance in desired social distance, so research is needed to identify additional contributors to problem gambling stigma to further inform anti-stigma measures. Future research might consider additional dimensions of problem gambling, or use different measures, given that those used in this study were developed for different mental illnesses and may not optimally explain problem gambling stigma. Future research could also overcome some limitations of the current study. These include using a panel rather than a random population sample, which may have introduced bias. Measures were based on responses to vignettes. Although a commonly used method in stigma research (Link et al. 2004), including for problem gambling (Horch and Hodgins 2008; Dhillon et al. 2011), results are highly dependent on how accurately each vignette captured the condition it represented. All vignettes included only a male protagonist; therefore the results may not generalise to women. Any research into stigma may be subject to social desirability bias. However, given that a moderate level of public stigma associated with problem gambling was revealed, any bias may be low and probably errs on the side of underestimation.

This paper is the first to study the stigma of problem gambling in non-university samples, for which no previous concepts or theories exist. The relationships found in this study generally align with those found in the mental illness stigma literature. Based on these findings, we tentatively suggest that concepts and theories from the mental illness stigma literature are a promising basis on which to base future studies on the stigma of problem gambling.

## Conclusion

The public stigma of problem gambling has deleterious effects by undermining the mental health of stigmatised individuals and posing a major barrier to problem acknowledgement, disclosure, treatment-seeking and recovery. Nevertheless, efforts to reduce the societal stigma attached to problem gambling have generally been minimal, although they are much needed and are far less advanced than those for many other physical and mental health conditions. Developing effective stigma reduction measures requires understanding why a condition is stigmatised, which in turn requires knowledge of how various characteristics of the condition are publicly perceived. This study advances this understanding in relation to problem gambling. It is the first to measure the public stigma associated with problem gambling using a general population sample, to examine how its various dimensions are publicly perceived, and to determine the contribution of these perceived dimensions to its stigmatisation. In addition to advancing knowledge of how problem gambling is viewed by the general public, and in relation to some other health conditions, this research has identified how five stigma-related dimensions of problem gambling contribute to its societal stigmatisation. As such, the findings provide some groundwork to help understand and reduce the public stigma of problem gambling.

## Appendix: Vignettes

### Problem Gambling (Adapted from Horch and Hodgins 2008)

Dan is a man who lives in your community. During the last twelve months, he has started to gamble more than his usual amount of money. He has even noticed that he needs to gamble much more than he used to in order to get the same feeling of excitement. Several times, he has tried to cut down, or stop gambling, but he can’t. Each time he has tried to cut down, he became agitated and couldn’t sleep, so he gambled again. He is often preoccupied by thoughts of gambling and gambles more to try to recover his losses. Dan has also lied to his family and friends about the extent of his gambling.

### Alcohol Use Disorder (Adapted from Link et al. 1999)

Peter is a man who lives in your community. During the last year Peter has started to drink more than his usual amount of alcohol. In fact, he has noticed that he needs to drink twice as much as he used to in order to get the same effect. Several times, he has tried to cut down, or stop drinking, but he can’t. Each time he has tried to cut down, he became very agitated, sweaty and he couldn’t sleep, so he took another drink.

### Schizophrenia (Adapted from Link et al. 1999)

Peter is a man who lives in your community. Up until a year ago, life was pretty okay for Peter. But then, things started to change. He thought that people around him were making disapproving comments and talking behind his back. Peter was convinced that people were spying on him and that they could hear what he was thinking. Peter lost his drive to participate in his usual work and family activities and retreated to his home, eventually spending most of his day in his room. Peter was hearing voices even though no one else was around. These voices told him what to do and what to think. He has been living this way for six months.

### Recreational Gambling (Developed for This Study)

Peter is a man who lives in your community. During the last year, Peter has started to gamble occasionally. He usually bets the same amount of money and never bets more than he intends. He stops gambling when he is losing and doesn’t lose very much money. He often goes long periods without gambling and does other leisure activities instead. He doesn’t find he misses gambling and he doesn’t think about gambling while he is away from it. Peter’s family and friends know that he sometimes gambles.

### Sub-clinical Distress Control (Adapted from Horch and Hodgins 2008)

John is a man who lives in your community. During the last year, life has been pretty okay for John. Most of the time he is pretty content, although he sometimes feels worried, a little sad, or has trouble sleeping at night. When things go wrong, he can usually handle the situation pretty well, although sometimes things bother him more than they should and he gets a bit down or annoyed. Nevertheless, most of the time he manages to keep his emotions under control and he is getting along pretty well.
